# Update on Coronary Angiography-Based Physiology Technologies

**DOI:** 10.5935/abc.20190140

**Published:** 2019-08

**Authors:** Alexandre Hideo-Kajita, Hector M. Garcia-Garcia, Evan Shlofmitz, Carlos M. Campos

**Affiliations:** 1 MedStar Health Research Institute - Medstar Cardiovascular Research Network (MHRI/MCRN), Hyattsville, Maryland - USA; 2 MedStar Washington Hospital Center, Washington, District of Columbia - USA; 3 Universidade de São Paulo - Faculdade de Medicina Hospital das Clinicas Instituto do Coração, São Paulo, SP - Brazil; 4 Hospital Israelita Albert Einstein - Cardiologia Intervencionista, São Paulo, SP - Brazil

**Keywords:** Coronary Artery Disease/physiopathology, Percutaneous Coronary Intervention, Angina, Stable, Coronary Angiography, Fractional Flow Reserve, Myocardial, Software/trends

From the early stages of percutaneous coronary intervention (PCI), Andreas Grüentzig had advocated that the direct measurement of the trans-stenotic pressure gradient after balloon PCI should be used as a marker of successful PCI.^1^ Since Grüentzig’s time, the physiologic assessment of coronary artery disease (CAD) has been tested and validated.^2^ Currently, fractional flow reserve (FFR) is the standard of care for the online assessment of CAD physiology, identifying hemodynamically significant lesions in stable angina patients.^3,4^

Albeit FFR is a relatively simple procedure, with a low complication rate, it comes with some intrinsic procedural risks and cost. Recently, non-hyperemic, resting index based physiology modalities have become an alternative to FFR but still require invasive assessment. Coronary angiography-based physiology technology was developed to overcome the intracoronary wiring and additional medication administration that were necessary with invasive physiology.^5^

Based on the principle of FFR, coronary angiography-based physiology technology incorporates computational power by combining the 3-dimensional (3D) meshing (i.e. virtual reconstructions) of the coronary artery and the use of computational fluid dynamics (CFD) as a surrogate marker of the antegrade coronary artery blood flow.^6^

## Computational fluid dynamics

The basis for CFD is derived from Navier-Stokes equations, a mathematical generalization of Euler’s flow of incompressible and frictionless fluids equation.^6^ In its current state, CFD now can compensate for 3 dimensionality and interactions in the non-perfect cylindrical shape of the coronary arteries.^6^ However, due to intrinsic cardiovascular physiology particularities, CFD cannot compensate for pulsatile blood flow effects; physiologic differences of coronary blood flow velocity in the proximal vs. distal segments of the vessel; and predictable loss of energy over a diseased vessel.^6-10^ Moreover, CFD still cannot address the high complexity interactions in vessel geometry that may lead to a chaotic vortex or turbulence formation and more importantly, the trans-lesional pressure drop.^6,10^

There are significant differences in the complex rheological properties of blood and normal blood flow along the coronary artery tree branches by itself that are not taken into account in these models. This includes the Newtonian versus Non-Newtonian fluid properties of blood that depend on the vessel diameter, the presence of a bifurcation, and slow blood flow shear stress effects (e.g. the non-Newtonian fluid property in that context).^6^ CFD simulation generalizes the differences of Newtonian and Non-Newtonian fluids properties by the assumption that large vessels can be approximated to a Newtonian fluid property with constant viscosity.^11,12^ Therefore, these data provide a consistent explanation of why these methods were not standardized to evaluate severe stenosis or antegrade blood flow in small vessels.^11^

## Computational time: Online vs. offline assessment

One of the major limitations for the clinical adoption of CFD in the online software is the computational time. The computation time required to estimate the antegrade blood flow in the 3D-mesh model using CFD considerably prolongs the procedure duration.^11^ In order to reduce the computational time and provide an online assessment of the vessel, most software developers substituted the CFD with mathematical coefficients.^11,12^ The impact of this substitution was studied by Collet et al. and demonstrated no significant difference between the results obtained using either method to estimate vessel blood flow.^13^

## Online coronary angiography-based physiology software

The development of online coronary angiography-based physiology software solutions occurred in parallel with different initiatives. Most commonly, their software solutions were tested and validated against invasive FFR, including Quantitative Flow Ratio (QFR), Cardiovascular Angiographic Analysis Systems-Vessel Fractional Flow Reserve (CAAS-vFFR) and Fractional Flow Reserve Derived From Coronary Angiography (FFR_angio_).^11,12,14^ Each software solution however used different metrics (i.e. pressure vs. TIMI frame count) and anatomic considerations (i.e. single vs. multi-vessel) to build the 3D-mesh and solve the CFD challenges of non-invasively predicting invasive FFR measurements, making a fair comparison among them unlikely.^11,12,14^

In its current state, the overall performance of online coronary angiography-based physiology was evaluated in a Bayesian meta-analysis showing a pooled sensitivity of 0.89, specificity of 0.90, the positive likelihood ratio of 9.3, the negative likelihood ratio of 0.13 and the summary area under the receiver-operating curve of 0.84 compared to invasive FFR.^13^ The individual characteristics of online coronary angiography-based physiology software solutions will be described below.

## Quantitative flow ratio (QFR)

**Figura 1:**
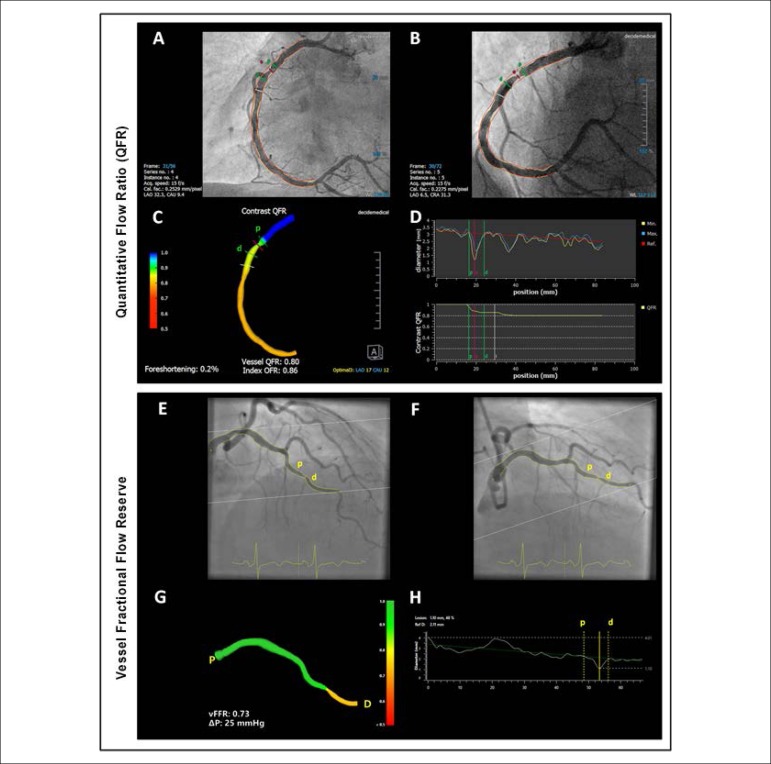
As imagens de A a D referem-se à artéria coronária direita (ACD) e avaliação da lesão pela Razão de Fluxo Quantitativo (QFR). As imagens E a H apresentam um vaso da artéria descendente anterior esquerda (ADA) com análise de lesão utilizando o Sistema de Análise Angiográfica Cardiovascular para Fluxo Fracionado de Reserva de vasos (SAAC-FFRv). Análise coronária quantitativa de duas projeções angiográficas ortogonais da ACD (A e B); Análise da QFR sobre a reconstrução tridimensional (3D) da ACD (C); Gráficos da QFR mostrando o diâmetro do vaso de referência proximal e distal da lesão e o ponto mais estreito da lesão (D). Análise do SAAC-FFRv mostrando projeções angiográficas ortogonais da ADA (E e F); Análise do SAAC-FFRv sobre a reconstrução em 3D da ADA (G); Gráficos de análise do SAAC-FFRv apresentando todo o diâmetro do vaso, marcando o diâmetro do vaso de referência proximal e distal da lesão, seguido pelo ponto mais estreito dentro da lesão (H). “P”: proximal; “D”: distal.

QFR (QAngioXA-3D prototype, Medis Medical Imaging System, Leiden, the Netherlands) is an angiography-based physiology software that uses the TIMI frame count of a single-vessel in two orthogonal views as the surrogate marker of blood flow to calculate the trans-lesional gradient ratio (Figure 1 A to D). In the latest reports, Favor II China trial, Xu B et al.^11^ showed a linear correlation (r) between invasive FFR and QFR (online assessment) of 0.86 (p < 0.001) with a mean agreement difference of -0.01±0.06 (p = 0.006).^11^ Spitaleri et al.^15^ reported that the absence of revascularization of non-culprit lesions in ST-elevation segment myocardial infarction (STEMI) patients with QFR ≤ 0.80 increased the risk of clinical events in this population (HR 2.3; CI 95%, 1.2-4.5; p = 0.01).^15^ Mejía-Rentería et al.^16^ highlighted that coronary microcirculatory dysfunction (CMD) affects the overall diagnostic performance of QFR.^16^ The QFR system has CE Mark and *ANVISA* clearance for clinical use. Clinical guidelines have not yet established the appropriate role of QFR in routine practice. Ongoing clinical trials including FAVOR III China (NCT03656848) may ultimately impact future guidelines.

## Cardiovascular angiographic analysis systems for vessel fractional flow reserve (CAAS-vFFR)

CAAS-vFFR, Pie Medical Imaging, Maastricht, The Netherlands is single-vessel, two orthogonal view angiography-based physiology software (Figure 1 E to H). The CAAS-vFFR validation study included 100 patients with intermediate lesions and stable CAD or non-STEMI. The CAAS-vFFR and FFR mean value were 0.84 ± 0.07 and 0.82 ± 0.08, respectively.^14^ The linear correlation of CAAS-vFFR vs. FFR was 0.89 (p < 0.001) and CASS-vFFR showed a high inter-observer correlation of 0.95 (p < 0.001). In addition, CAAS-vFFR diagnostic accuracy for lesions with FFR ≤ 0.80 was 0.93 (p < 0.001).^14^ CAAS-vFFR was the first angiography-based physiology system to receive Food and Drug Administration (FDA) *“USA 510(k) approval”* market clearance.

## Fractional flow reserve derived from coronary angiography (FFR_angio_)

Unlike QFR and CAAS-vFFR, the Fractional Flow Reserve Derived From Coronary Angiography (FFR_angio_, CathWorks Ltd., Kfar-Saba, Israel) reconstructs the entire coronary artery tree using 3 single-plane angiographic projections (at least) and the mean aortic pressure to calculate a virtual FFR mapping of the 3D-model.^12,17^ Fearon et al.^17^ performed a global, multi-center validation study of FFR_angio_ enrolling 301 all-comer patients (319 lesions).^17^ FFR_angio_ and invasive FFR measurements varied from 0.74-0.90 (median 0.83) and 0.5-0.97 (median 0.85), respectively. The coefficient of correlation between FFR_angio_ and invasive FFR was 0.80 (p < 0.001) and Bland-Altman’s confidence limits were between -0.14 and 0.12 (95%). For lesions with invasive FFR ≤ 0.80, FFR_angio_ (per vessel) demonstrated the sensitivity of 0.94, the specificity of 0.91 and area under the curve of 0.94. The overall FFR_angio_ diagnostic accuracy was 0.92 and 0.87 for invasive FFR values between 0.75-0.85.^17^ Finally, the inter-observer consistency of agreement between the methods was 0.96 (p < 0.001).^12^

## Physiology assessment cost-effectiveness

A recurrent criticism of the routine use of invasive physiologic assessment (i.e. FFR) of CAD in the cardiac catheterization laboratory is the additional procedural cost.^18,19^ For the non-invasive angiography-based physiology methods, cost-effectiveness data needs to be further investigated. This technology involves upfront institutional hardware and software costs, rather than a specific case-by-case cost of an invasive wire.

## Impact of physiologic lesion assessment on clinical outcomes

Currently, with contemporary stents, target lesion failure (TLF, a composite of cardiac death, target vessel MI, or ischemia-driven target lesion revascularization - TLR) rates are similar (i.e. 4.0% to 6.0% at 12 months) to the rate of combined endpoint in the group of patients in whom PCI was deferred on the basis of FFR (i.e. overall unplanned revascularization of 5.0% at 12 months).^20^ Thus, the rate of composite events for the treatment or deferral of PCI are similar, perhaps limiting the appropriate utilization of FFR for informing PCI decisions.^20-22^ This needs to be further investigated in a contemporary clinical trial using 2^nd^ generation drug-eluting stents (2G-DES).

## Conclusion

The majority of angiography-based physiology software solutions are currently available for research only. Clinical trials demonstrating clinical feasibility and reproducibility with a significant impact on clinical outcomes are needed. However, real-world studies are also needed to evaluate the reliability, integration and cost-effectiveness of these technologies in a clinical catheterization laboratory, since the prevalence of ischemic lesions in most studies is limited (i.e. 17% to 43%).^17^ The angiography-based physiology technologies have great potential, but still need to be observed with a word of caution and the impact of these technologies remains unknown.
